# Cognitive factors that predict on-sight and red-point performance in sport climbing at youth level

**DOI:** 10.3389/fpsyg.2022.1012792

**Published:** 2022-11-30

**Authors:** Antonia Ioana Vasile, Monica Stănescu, Florin Pelin, Roxana Bejan

**Affiliations:** ^1^Doctoral School, National University of Physical Education and Sport, Bucharest, Romania; ^2^Sport and Motor Performance, National University of Physical Education and Sport, Bucharest, Romania; ^3^Teaching Department, National University of Physical Education and Sport, Bucharest, Romania

**Keywords:** sports climbing, elite climbers, youth climbing, cognitive training, image generation

## Abstract

**Introduction:**

The ascent of a route can be defined as being climbed on-sight or red-point. Climbing performance is measured by the grade of the personal best route that the athlete has ever climbed.

**Methodology:**

The study examined 17 youth climbers (10 male and 7 female). The inclusion criteria were age (less than 20 years), a minimum of three sessions per week, a minimum 7a climbing grade and participation in national or international competitions. We used the Cognitrom battery and applied tests measuring spatial orientation and reactivity.

**Results:**

Climbing experience explained 42.7% of the variance of on-sight performance, and 49.5% of the variance of red-point performance. Image generation has a negative on both on-sight and red-point performance, lowering the prediction with 0.5% for on-sight climbing and with 1.5% for red-point climbing.

**Discussion:**

Experience can predict climbing performance with a better prediction for red-point performance than on-sight with almost 7%. A high level of image generation ability can lead to viewing more approaches for passing the crux, but in a moment of physical and mental breakdown, can lead to failure. Red-pointing is less demanding than on-sight from physiological and psychologycal points of view. On-sight climbing requires greater levels of cognitive skills, such as route intepretation strategies, spatial orientation, motric memory, problem-solving skills, but also greater levels of psychological skills such as stress management, risk management, coping anxiety.

## Introduction

The popularity of indoor sport climbing as a recreational or competitive activity has increased in the recent years (Booth et al., 1999). Climbing is described as a vigorous activity that demands muscular power and strength, flexibility and aerobic endurance (Kascenska et al., 1992). It also increases cardiorespiratory fitness and muscle endurance (Mermier et al., 1997). Williams et al. (1978) explain that climbing produces a specialized type of fitness that improves climbing performance but not general fitness. It has been highlighted that climbing ability is dependent on experience and technique but also physiological and psychological skills (Birkett, 1988). Climbing involves ascending routes on different artificial or rock surfaces, indoors or outdoors (Draper et al., 2011a).

Sport climbing is an emergent discipline that was included for the first time in the 2020 Olympic Games. It is also growing as a competitive sport and has attracted a lot of interest for researchers to understand the factors that lead to performance. The ascent of a route can be defined as being climbed on-sight or red-point (Limonta et al., 2020). A route is climbed *on-sight* when the athlete completes it without any fall and without any previous practice or knowledge about the holds or the plan. The athlete is not allowed to watch other athletes climbing that route or to obtain additional information about grips, movements or the approach plan (Schweizer and Furrer, 2007). The climber is allowed to visually inspect the route from the ground. On-sight climbing is considered the purest, the more challenging because it demands greater physiological and psychological engagement (Draper et al., 2008). If the route is not covered at the first attempt but is completed after practice, it is termed a *red-point* (Draper et al., 2011b).

There is a difference between climbing performance as a personal best route climbed by an athlete and competitive performance as a social comparison between athletes. The format of a competition dictates an intermittent activity pattern and anecdotal evidence indicates that bouldering requires considerable strength and power in the fingers and forearms, with primarily anaerobic energy (Horst, 2003; Watts, 2004). Determining activity patterns for intermittent activity is important because this process can establish the different movement characteristics between climbing during training and climbing during a competition (Rhea et al., 2006). National and international competitions are organized for three disciplines: lead (climbing with rope protection), bouldering (lower heights of the routes with mattress floor protection) and speed (maximum climbing speed on a standardized route) (Lutter et al., 2021).

Climbing performance is measured with an international scale assessing the difficulty of the route completed. Climbing performance can be measured on several scales: Yosemite Decimal System (YDS) used in the United States, the French sport scale used in Europe, the British technical grading scale (for traditional routes), the Ewbank scale used in Australia, New Zealand and South Africa, and the UIAA scale primarily used in Germany, Austria, Switzerland, Czech Republic, Slovakia and Hungary (Draper et al., 2015). The French scale for grading the routes implies numbers from 4 to 9, which are subdivided into the letters a, a+, b, b+, c, c+. Athletes who achieve at least grade 7a (according to the French rating scale) are considered experts or elite (Watts, 2004; Ascii et al., 2007).

The literature postulates the idea that there are certain cognitive skills such as problem-solving skills, movement memory, and visualization that could be better predictors of sports performance compared to biomechanical or physiological variables (Morrison and Schöffl, 2007; Jones and Sanchez, 2017). Gnostic functions allow the climber to understand the movement to be performed in terms of spatial orientation; the climber must also have the mental ability to imagine, plan, and anticipate the movement. Climbers must use adequate strength as well as suppress excessive movements and rhythmicity. Naderi et al. (2018) note that, in addition to physical tasks, bouldering offers cognitive challenges for planning and discovering all possible permutations of movements (from the optimal to the least possible ones) to get from the starting grips to the top. It seems that climbing may rival any other sport, requiring high levels of attention and body control. If a simultaneous cognitive task interferes with cyclical and near-automatic activity (such as walking, according to the study by Yogev-Seligmann et al. (2010), we expect much greater interference for physical activity that requires increased attention (like climbing, in which the athlete is in a continuous postural instability).

Limonta et al. (2020) talk about two cognitive essential skills that optimize climbing performance: route interpretation ability and movement sequence recall, both of them being trainable skills. In on-sight climbing, climbers need to develop route interpretation strategies before the ascent and increase their problem-solving ability. Route interpretation strategies and problem-solving ability can be optimized and will lead to better climbing (Boschker et al., 2002). Because of that, during competition and training, climbers visually inspect the route from the ground (doing the route preview) in order to understand and visualize the optimal sequence of moves they will need to climb (Limonta et al., 2020). Scientists (Sanchez et al., 2012) consider that route preview errors are a major reason for falling. On the other hand, in red-point climbing, the ability to memorize (retaining information acquired during the previous attempts) is also a crucial skill (Sanchez et al., 2012). Exploratory behavior (influenced by past experiences) and mnemonic skills are potential indicators for learning and performance in climbing. Climbers use route preview to plan the order of climbing moves, determine the best plan to ascend, improve speed and efficiency and find the best rest places during the climb (Boschker et al., 2002). But the route preview depends on past experiences. It has been demonstrated that advanced climbers are able to accurately perceive the maximum distance they can reach and program their movements accordingly, whereas beginners underestimate the maximum reaching place (Boschker et al., 2002). More advanced climbers are trained to perform a wider range of technical movements (Sanchez et al., 2019). In summary, researchers support the importance of certain cognitive processes such as memory, attention, reaction time and spatial orientation for climbing performance.

The aim of this study is to identify how cognitive factors (spatial orientation and reaction time) can influence performance in youth elite sport climbing, with an emphasis on the differences between on-sight and red-point climbing.

## Methodology

### Participants

The study was conducted on 17 male (58.82%) and female (41.18%) climbers aged between 13 and 19 years (M = 16.59 years; DS = 2.00). *Age* was among the inclusion criteria: all climbers are under 20 years old so that they can participate in youth competitions: aged between 13 and 14 years-4 athletes (23.52%), aged between 15 and 16 years-4 athletes (23.52%) and aged between 17 and 19 years-9 athletes (52.94%). Other inclusion criteria were: a *training frequency* of at least three sessions per week, the minimum climbed grade of *7a* (on-sight or red-point, bouldering or lead, on artificial walls or on rocks) and inclusion in the *national federal team* with active participation in national and international competitions. The climbers participated in at least one national or international competition in the past year before the study. In addition, they were tested in the off-season, in the pre-competition stage, just before the start of the world and European cups. All the climbers can participate in youth competitions, but 10 frequently compete in international competitions, while 7 compete in national competitions only. The inclusion criteria were very drastic, trying to analyze the entire elite youth climbers from Romania. We analyzed the entire Romanian youth climbing team that competes in National and International competitions. Climbing is an underdeveloped sport in Romania, with a few people climbing as a recreational activity compared to other European climbers [such as France where pupils can practice climbing at the physical education class (Attali and Saint-Martin, 2016)]. There are 31 sports clubs in Romania, but with active participation to National competitions the athletes come only from 5.^1^ In a statistical analysis from 2021,^2^ at the youth level, there were affiliated 60 athletes, from which 48 of them participated in National competitions. When we added the inclusion criterion of climbing above 7a, we remained with 17 athletes, which can be considered a representative sample.

In terms of on-sight climbing performance, the athletes vary from 6c to 8b, and for red-point climbing performance, from 7a to 9a. We used the Frech scale to measure climbing performance. Athletes climb 3 to 7 times a week (*M* = 4.29, DS = 1,312), with a duration ranging from 2 to 4 h (*M* = 2.85, DS = 0.52). Their sports experience (number of years the athlete practices climbing) varies from 1 to 12 years (*M* = 6.94, DS = 3.01) and their competitive experience (number of years the athlete participates in internal or external competitions) varies from 0.5 to 11 years (*M* = 4.941, DS = 3.3).

Each athlete who was over 18 years old gave written consent to participate in the study. For minors, we collected written consent from their parents, but also verbal consent from their climbing coaches for evaluation. All participants received a full explanation about the study design and objectives and how the results might help them find out what their weaknesses were and how their climbing could be improved.

The studies involving human participants were reviewed and approved by the Ethical Committee of the National University of Sport and Physical Education, Bucharest, Romania. Written informed consent to participate in this study was provided by the participants’ legal guardian/next of kin.

The license for applying the cognitive tests belonged to one of the researchers.

### Instruments and variables

**Figure 1:**
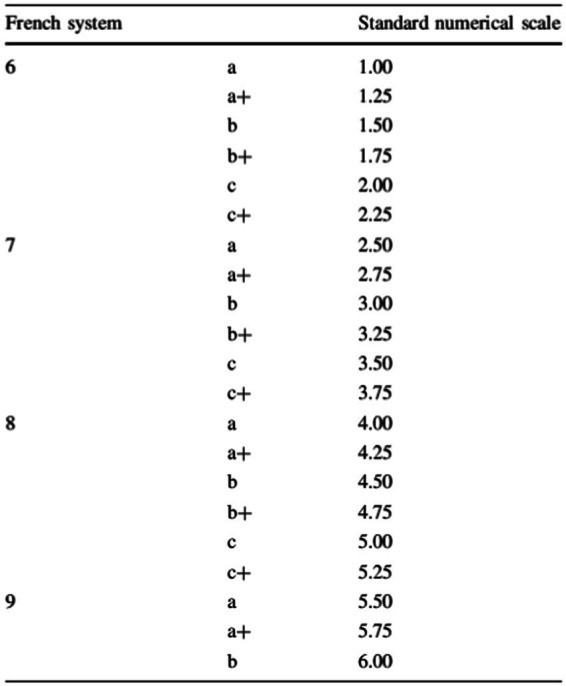
Conversion scale for standardizing climbing performance (Watts et al., 1993).

The factual variables measured were: age, sports experience, competitive experience, climbing performance (divided into on-sight performance and red-point performance, and then converted into a standard numerical scale taken from the literature) (Watts et al., 1993; Figure 1).

Given the directions highlighted by the literature, we decided to perform the assessment using the Cognitrom battery of standardized tests: *spatial skills* (with 3 subtests that measure: *mental image-transformation* test, *spatial orientation* test and *image generation* test) and *reaction speed* (with 3 subtests: *simple reaction* time, *choice reaction* time and *memory access reaction* time). We decided to use only these tests because the assessment lasted about 1 h, which is why the participants were prone to mental overload. The results were received by the assessor through a psychological evaluation report. The result of each test was expressed on a value scale of 1 to 5 (very weak, weak, medium, good and very good), but also as a raw score. We interpreted the raw results because these were parametric variables.

The mental image-transformation test analyses the subject’s ability to mentally represent some images and then rotate them in their mind. The spatial orientation test measures the subject’s ability to analyze a visual field from different points of view. The image generation test measures the subject’s ability to analyze a series of images and then combine them so that they form in their mind a new image and identify it in the shortest time possible.

The simple time reaction measures the speed that a subject needs to respond to the appearance of a visual stimulus. Choice reaction time measures the speed that a subject needs to choose from some alternatives and it is in direct relation to decisional capacity. The memory access reaction time measures the speed that a subject needs to access information from their memory so it measures the time that the subject needs to analyze their memory and decide whether the information they see in the present is or not in their mind.

We used a cross-sectional study design.

### Statistical analysis

Due to the existence of parametric variables, we used Pearson correlation to assess the relationships between variables. Moreover, we wanted to test whether the assessed cognitive variables could predict climbing performance. We used the ANOVA analysis to check whether the predictive model was statistically relevant. After confirmation, we predicted both on-sight and red-point performance based on the assessed cognitive variables. We used the Backward statistical method that would eventually provide the correct statistical model by trying in turn all possible models, including the inserted independent variables and eliminating one by one those that were statistically irrelevant.

The first experimental design **(A)** was: dependent variable was on-sight-performance with independent variables were all the cognitive scales adding experience (climbing experience and competitive experience) and age. The second experimental design **(B)** was: dependent variable was red-point-performance with independent variables were all the cognitive scales adding experience (climbing experience and competitive experience) and age.

## Results

The descriptive statistic with means, standard deviation, skewness and kurtosis for all measured variables can be identified in Table 1. We can see that all variables except memory access reaction time have a normal distribution: the absolute value of skewness is less than 3 and the absolute value of kurtosis is less than 8 (Kline, 2011).

**Table 1:** Descriptive analysis.

	Minimum	Maximum	Mean	Std. Deviation
Age	13	19	16.59	2.002
Climbing experience	1	12	6.94	3.010
Competitive experience	0.5	11.0	4.941	3.3019
On-sight performance	2.00	4.50	3.0441	0.77174
Red-point performance	2.50	5.50	3.7647	0.89474
Mental images	10	18	13.65	2.668
Spatial orientation	12	18	15.65	2.060
Image generation	7	13	10.65	2.120
Simple reaction time	221	451	276.24	62.057
Choice reaction time	633	1,600	1052.06	315.774
Memory access reaction time	761	2,413	1101.88	375.052

We tested using Pearson correlation the relationships between variables and the results can be seen in Tables 2, 3.

**Table 2:** Correlation analysis.

	Correlations						
		Age	Climbing experience	Competitive experience	On-sight performance	Red-point performance	Mental images
Age	*R*	1	0.110	0.029	0.225	0.117	0.369
	*p*-value		0.675	0.911	0.386	0.655	0.145
Climbing experience	*R*	0.110	1	0.858^**^	0.681^**^	0.726^**^	0.168
	*p*-value	0.675		0.000	0.003	0.001	0.518
Competitive experience	*R*	0.029	0.858^**^	1	0.648^**^	0.704^**^	−0.010
	*p*-value	0.911	0.000		0.005	0.002	0.971
On-sight performance	*R*	0.225	0.681^**^	0.648^**^	1	0.910^**^	0.213
	*p*-value	0.386	0.003	0.005		0.000	0.412
Red-point performance	*R*	0.117	0.726^**^	0.704^**^	0.910^**^	1	0.166
	*p*-value	0.655	0.001	0.002	0.000		0.524
Mental images	*R*	0.369	0.168	−0.010	0.213	0.166	1
	*p*-value	0.145	0.518	0.971	0.412	0.524	
Spatial orientation	*R*	0.569^*^	−0.135	−0.316	−0.167	−0.099	0.340
	*p*-value	0.017	0.606	0.217	0.523	0.706	0.182
Image generation	*R*	0.479	−0.346	−0.543^*^	−0.401	−0.557^*^	0.120
	*p*-value	0.052	0.173	0.024	0.111	0.020	0.646
Simple reaction time	*R*	−0.070	0.094	0.022	−0.141	−0.118	−0.163
	*p*-value	0.791	0.720	0.934	0.588	0.652	0.531
Choice reaction time	*R*	0.167	0.146	0.145	0.121	0.010	−0.059
	*p*-value	0.521	0.575	0.579	0.643	0.971	0.821
Memory access reaction time	*R*	−0.203	0.162	0.260	0.380	0.325	−0.329
	*p*-value	0.435	0.535	0.314	0.132	0.203	0.197

*Correlation is significant at the 0.05 level (2-tailed). **Correlation is significant at the 0.01 level (2-tailed).

**Table 3:** Correlation analysis.

		Correlations				
		Spatial orientation	Image generation	Simple reaction time	Choice reaction time	Memory access reaction time
Age	*R*	0.569^*^	0.479	−0.070	0.167	−0.203
	*p*-value	0.017	0.052	0.791	0.521	0.435
Climbing experience	*R*	−0.135	−0.346	0.094	0.146	0.162
	*p*-value	0.606	0.173	0.720	0.575	0.535
Competitive experience	*R*	−0.316	−0.543^*^	0.022	0.145	0.260
	*p*-value	0.217	0.024	0.934	0.579	0.314
On-sight performance	*R*	−0.167	−0.401	−0.141	0.121	0.380
	*p*-value	0.523	0.111	0.588	0.643	0.132
Red-point performance	*R*	−0.099	−0.557^*^	−0.118	0.010	0.325
	*p*-value	0.706	0.020	0.652	0.971	0.203
Mental images	*R*	0.340	0.120	−0.163	−0.059	−0.329
	*p*-value	0.182	0.646	0.531	0.821	0.197
Spatial orientation	*R*	1	0.356	0.151	−0.413	−0.428
	*p*-value		0.161	0.563	0.099	0.086
Image generation	*R*	0.356	1	0.198	0.219	−0.092
	*p*-value	0.161		0.445	0.398	0.724
Simple reaction time	*R*	0.151	0.198	1	−0.082	−0.112
	*p*-value	0.563	0.445		0.755	0.668
Choice reaction time	*R*	−0.413	0.219	−0.082	1	0.441
	*p*-value	0.099	0.398	0.755		0.077
Memory access reaction time	*R*	−0.428	−0.092	−0.112	0.441	1
	*p*-value	0.086	0.724	0.668	0.077	

*Correlation is significant at the 0.05 level (2-tailed).

From the correlation analysis, we can conclude the following. On-sight performance is positively correlated with: climbing experience (*p* = 0.003; *R* = 0.681; *R*^2^ = 0.463), competitive experience (*p* = 0.005; R = 0.648; R^2^ = 0.214), red-point performance (*p* = 0.000; *R* = 0.910; *R*^2^ = 0.828). Red-point performance is positively correlated with: climbing experience (*p* = 0.01; *R* = 0.726; *R^2^* = 0.527), competitive experience (*p* = 0.002; *R* = 0.704; *R*^2^ = 0.495), on-sight performance (*p* = 0.000; *R* = 0.910; *R*^2^ = 0.828) and negatively correlated with image generation (*p* = 0.020; *R* = −0.557; *R*^2^ = 0.310). We can conclude from this analysis that experience positively correlates with performance in climbing.

**Figure 2:**
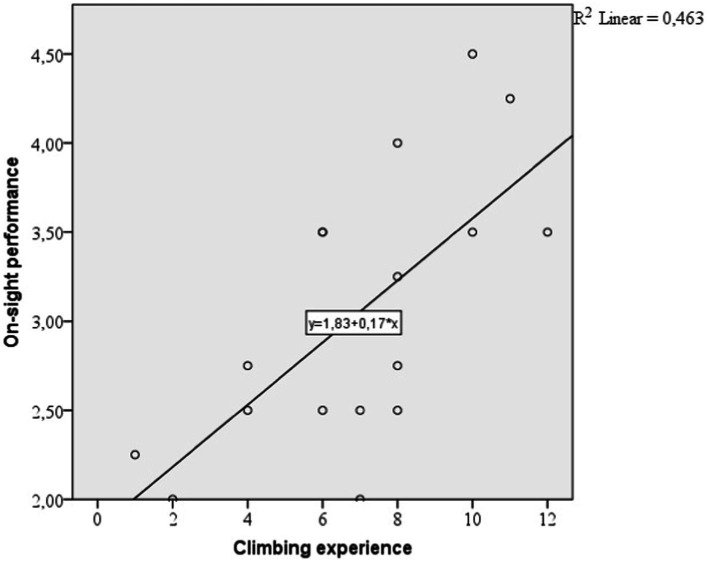
Linear regression between climbing experience and on sight performance.

**Figure 3:**
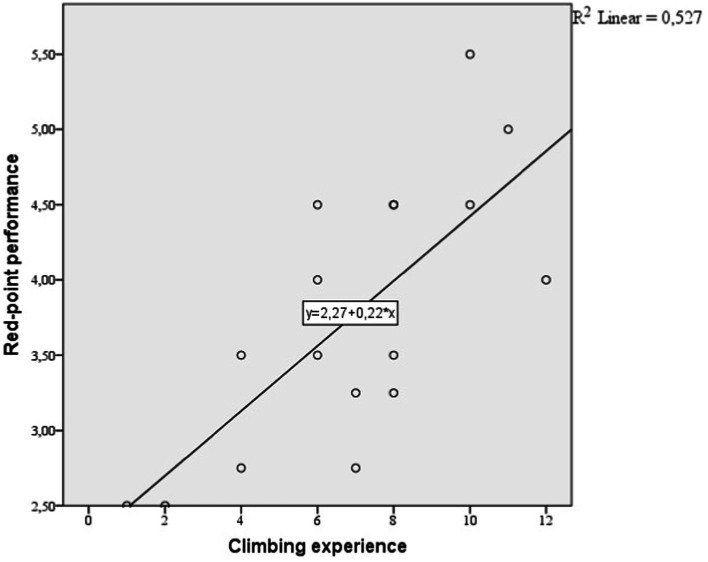
Linear regression between climbing experience and red-point performance.

Further, we wanted to predict the two types of performance by experience using a linear regression analysis. On-sight performance is predicted by climbing experience with a prediction of 42.7% (*p* = 0.03; R = 0.681; *R*^2^ = 0.463; adjusted *R*^2^ = 0.427, Beta = 0.175). Red-point performance is predicted by climbing experience with a prediction of 49.5% (*p* = 0.01; *R* = 0.726; *R*^2^ = 0.527; adjusted *R*^2^ = 0.495, Beta = 0.216). The linear prediction can be illustrated in Figures 2, 3.

In order to see the influence of the cognitive variables on on-sight performance, we first tested for multicollinearity. Because the VIF value for competitive experience was above 5 (VIF = 7.995), we had to exclude this variable. The VIF values for the dependent variable on-sight after excluding competitive experience are seen in Table 4.

**Table 4:** VIF values for on-sight performance.

Model	Coefficients^a^	
	Collinearity Statistics	
	Tolerance	VIF
(Constant)		
Age	0.360	2.780
Climbing experience	0.646	1.548
Spatial orientation	0.357	2.799
Image generation	0.478	2.094
Simple reaction time	0.784	1.276
Choice reaction time	0.469	2.133
Memory access reaction time	0.697	1.435

a Dependent variable: on-sight performance.

We did the same algorithm for verifying the multicollinearity for red-point performance as dependent variable and had to exclude the competitive experience (VIF = 8.202). The VIF values for the dependent variable on-sight after excluding competitive experience are seen in Table 5.

**Table 5:** VIF values for red-point performance.

Model	Coefficients^a^	
	Collinearity Statistics	
	Tolerance	VIF
(Constant)		
Age	0.360	2.780
Climbing experience	0.591	1.691
Mental images	0.686	1.457
Spatial orientation	0.344	2.904
Image generation	0.472	2.119
Simple reaction time	0.716	1.396
Choice reaction time	0.466	2.146
Memory access reaction time	0.630	1.586

a Dependent variable: Red-point performance.

Further, we performed two multiple linear regression analyzes, first with on-sight performance as the dependent variable and second with red-point performance as the dependent variable. We used the Backward statistical method, which would eventually provide the correct statistical model by trying in turn all possible models, including the inserted independent variables and eliminating one by one those that were statistically irrelevant. Using this method helped us because we introduced all measured variables without the ones excluded before (age, climbing experience, mental imaging, spatial orientation, image generation, simple time reaction, choice reaction time) as independent and the program provided the most relevant model.

### Predicting on-sight performance (A.)

Table 6 explains the model summary generated to predict the dependent variable. The highest adjusted R^2^ = 0.499, so the model explains almost 50% of the variance of on-sight performance (model 4).

**Table 6:** Model summary for predicting on-sight performance.

Model	Model summary^h^				
	*R*	*R* square	Adjusted *R* square	Std. error of the estimate	Durbin–Watson
1	0.798^a^	0.637	0.355	0.61977	
2	0.798^b^	0.637	0.419	0.58812	
3	0.796^c^	0.633	0.466	0.56389	
4	0.790^d^	0.625	0.499	0.54605	
5	0.764^e^	0.583	0.487	0.55265	
6	0.703^f^	0.494	0.422	0.58681	
7	0.681^g^	0.463	0.427	0.58397	1.569

a Predictors: (Constant), choice reaction time, mental images, simple reaction time, climbing experience, age, image generation, spatial orientation. b. Predictors: (Constant), choice reaction time, mental images, climbing experience, age, image generation, spatial orientation. c. Predictors: (Constant), mental images, climbing experience, age, image generation, spatial orientation. d. Predictors: (Constant), climbing experience, age, image generation, spatial orientation. e. Predictors: (Constant), climbing experience, age, image generation. f. Predictors: (Constant), climbing experience, image generation. g. Predictors: (Constant), climbing experience. h. Dependent Variable: on-sight performance.

We also had to check the ANOVA analysis for the relevance of the model (*p* = 0.030). The result can be seen in Table 7. We can see in Table 7 that models 3, 4, 5 and 6 are efficient in predicting the dependent variable. The *F* value will explain the most relevant one.

**Table 7:** ANOVA for predicting on-sight performance.

Model		ANOVA^a^				
		Sum of squares	*df*	Mean square	*F*	Sig.
1	Regression	6.072	7	0.867	2.258	0.127^b^
	Residual	3.457	9	0.384		
2	Regression	6.071	6	1.012	2.925	0.065^c^
	Residual	3.459	10	0.346		
3	Regression	6.032	5	1.206	3.794	0.030^d^
	Residual	3.498	11	0.318		
4	Regression	5.951	4	1.488	4.990	0.013^e^
	Residual	3.578	12	0.298		
5	Regression	5.559	3	1.853	6.067	0.008^f^
	Residual	3.971	13	0.305		
6	Regression	4.709	2	2.354	6.837	0.008^g^
	Residual	4.821	14	0.344		

a Dependent Variable: on-sight performance. b. Predictors: (Constant), choice reaction time, mental images, simple reaction time, climbing experience, age, image generation, spatial orientation. c. Predictors: (Constant), choice reaction time, mental images, climbing experience, age, image generation, spatial orientation. d. Predictors: (Constant), mental images, climbing experience, age, image generation, spatial orientation. e. Predictors: (Constant), climbing experience, age, image generation, spatial orientation. f. Predictors: (Constant), climbing experience, age, image generation. g. Predictors: (Constant), climbing experience, image generation.

The F value is maximum in model 6 (with *p* value = 0.08). In Table 6 we see that this model explains 42.2% of the variance of the on-sight performance (*R* = 0.703; *R*^2^ = 0.494; adjusted *R*^2^ = 0.422). The Cohen’s coefficient for the regression analysis predicting on-sight performance is 0.988, which corresponds for large effect size. We also checked for Durbin Watson value (=1.569) to be in the normal range.

Table 8 shows the standardized and non-standardized regression coefficients together with the t tests, which have helped us develop the multiple regression equation for the dependent variable.

**Table 8:** Coefficients for predicting on-sight performance.

Model		Coefficients^a^				
		Unstandardized coefficients		Standardized coefficients	*t*	Sig.
		*B*	Std. error	Beta		
6	(Constant)	2.675	0.982		2.725	0.016
	Climbing experience	0.158	0.052	0.616	3.038	0.009
	Image generation	−0.068	0.074	−0.187	−0.925	0.049
	Climbing experience	0.175	0.049	0.681	3.598	0.003

a Dependent variable: On-sight performance.

From Table 8 we can identify the Beta coefficients for the predicting variables. We notice that both variables are statistically significant (*p* < 0.05). The Beta coefficient for climbing experience is positive (Beta = 0.158) which is concordant with the linear regression, but the Beta coefficient for image generation is negative (Beta = −0.068). This result explains that when image generation is higher, the on-sight performance is lower.

**Figure 4:**
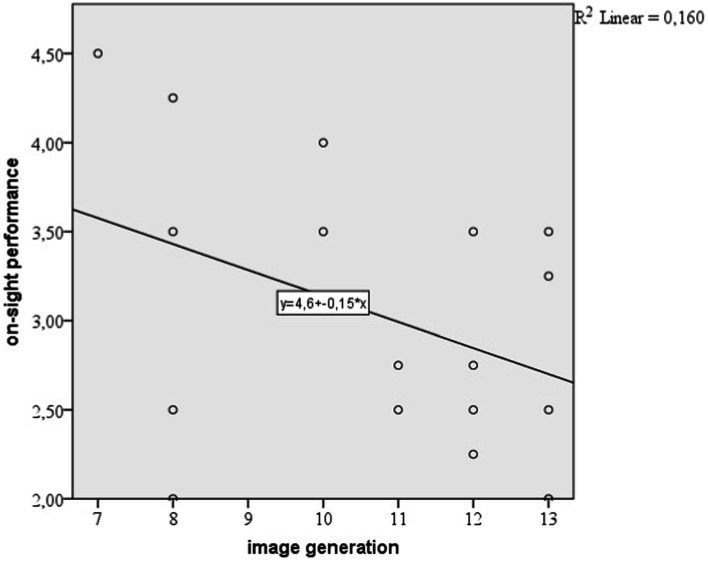
Scatterplot explaining simple linear regression between image generation and on sight performance.

We can see the same result on the scatterplot showed in Figure 4, that is a simple regression model where the independent variable is image generation and the dependent variable is on-sight performance.

### Predicting red-point performance (B.)

Table 9 explains the model summary generated to predict the dependent variable. The highest adjusted R^2^ = 0.633, so the model explains 63.6% of the variance of red-point performance. Also, the Durbin Watson coefficient is in the normal range value.

**Table 9:** Model summary for predicting red-point performance.

Model	Model summary^g^				
	*R*	*R* square	Adjusted *R* square	Std. error of the estimate	Durbin–Watson
1	0.840^a^	0.705	0.476	0.64789	
2	0.839^b^	0.705	0.527	0.61515	
3	0.839^c^	0.704	0.570	0.58704	
4	0.839^d^	0.703	0.605	0.56258	
5	0.838^e^	0.702	0.633	0.54198	
6	0.796^f^	0.633	0.480	0.57952	1.505

a Predictors: (Constant), choice reaction time, mental images, simple reaction time, climbing experience, age, image generation, spatial orientation. b. Predictors: (Constant), mental images, simple reaction time, climbing experience, age, image generation, spatial orientation. c. Predictors: (Constant), mental images, simple reaction time, climbing experience, age, image generation. d. Predictors: (Constant), simple reaction time, climbing experience, age, image generation. e. Predictors: (Constant), climbing experience, age, image generation. f. Predictors: (Constant), climbing experience, image generation. g. Dependent Variable: red-point performance.

We also had to check the ANOVA analysis for the relevance of the model (*p* = 0.030). The result can be seen in Table 10. We can see in Table 10 that models 2, 3, 4, 5 and 6 are efficient in predicting the dependent variable. The *F* value will explain the most relevant one.

**Table 10:** ANOVA for predicting red-point performance.

Model		ANOVA^a^				
		Sum of squares	*df*	Mean square	*F*	Sig.
1	Regression	9.031	7	1.290	3.073	0.060^b^
	Residual	3.778	9	0.420		
2	Regression	9.025	6	1.504	3.975	0.027^c^
	Residual	3.784	10	0.378		
3	Regression	9.018	5	1.804	5.234	0.011^d^
	Residual	3.791	11	0.345		
4	Regression	9.011	4	2.253	7.118	0.004^e^
	Residual	3.798	12	0.316		
5	Regression	8.990	3	2.997	10.202	0.001^f^
	Residual	3.819	13	0.294		
6	Regression	8.107	2	4.054	12.070	0.001^g^
	Residual	4.702	14	0.336		

a Dependent Variable: red-point performance. b. Predictors: (Constant), choice reaction time, mental images, simple reaction time, climbing experience, age, image generation, spatial orientation. c. Predictors: (Constant), mental images, simple reaction time, climbing experience, age, image generation, spatial orientation. d. Predictors: (Constant), mental images, simple reaction time, climbing experience, age, image generation. e. Predictors: (Constant), simple reaction time, climbing experience, age, image generation. f. Predictors: (Constant), climbing experience, age, image generation. g. Predictors: (Constant). climbing experience, image generation.

The F value is maximum in model 6 (with *p* value = 0.01). In Table 9 we see that this model explains 48% of the variance of the red-point performance (R = 0.796; R^2^ = 0.633; adjusted R^2^ = 0.48). The Cohen’s coefficient for the regression analysis predicting red-point performance is 1.313, which corresponds for large effect size.

Table 11 shows the standardized and non-standardized regression coefficients together with the t tests, which have helped us develop the multiple regression equation for the dependent variable.

**Table 11:** Coefficients for predicting red-point performance.

		Coefficients^a^				
		Unstandardized coefficients		Standardized coefficients		
Model		*B*	Std. error	Beta	*t*	Sig.
6	(Constant)	4.079	0.969		4.207	0.001
	Climbing experience	0.180	0.051	0.605	3.506	0.003
	Image generation	−0.147	0.073	−0.348	−2.015	0.044

a Dependent variable: red-point performance.

From Table 11 we can identify the Beta coefficients for the predicting variables. We notice that both variables are statistically significant (*p* < 0.05). The Beta coefficient for climbing experience is positive (Beta = 0.180) which is concordant with the linear regression, but the Beta coefficient for image generation is negative (Beta = −0.147). This result explains that when image generation is higher, the red-point performance is lower.

**Figure 5:**
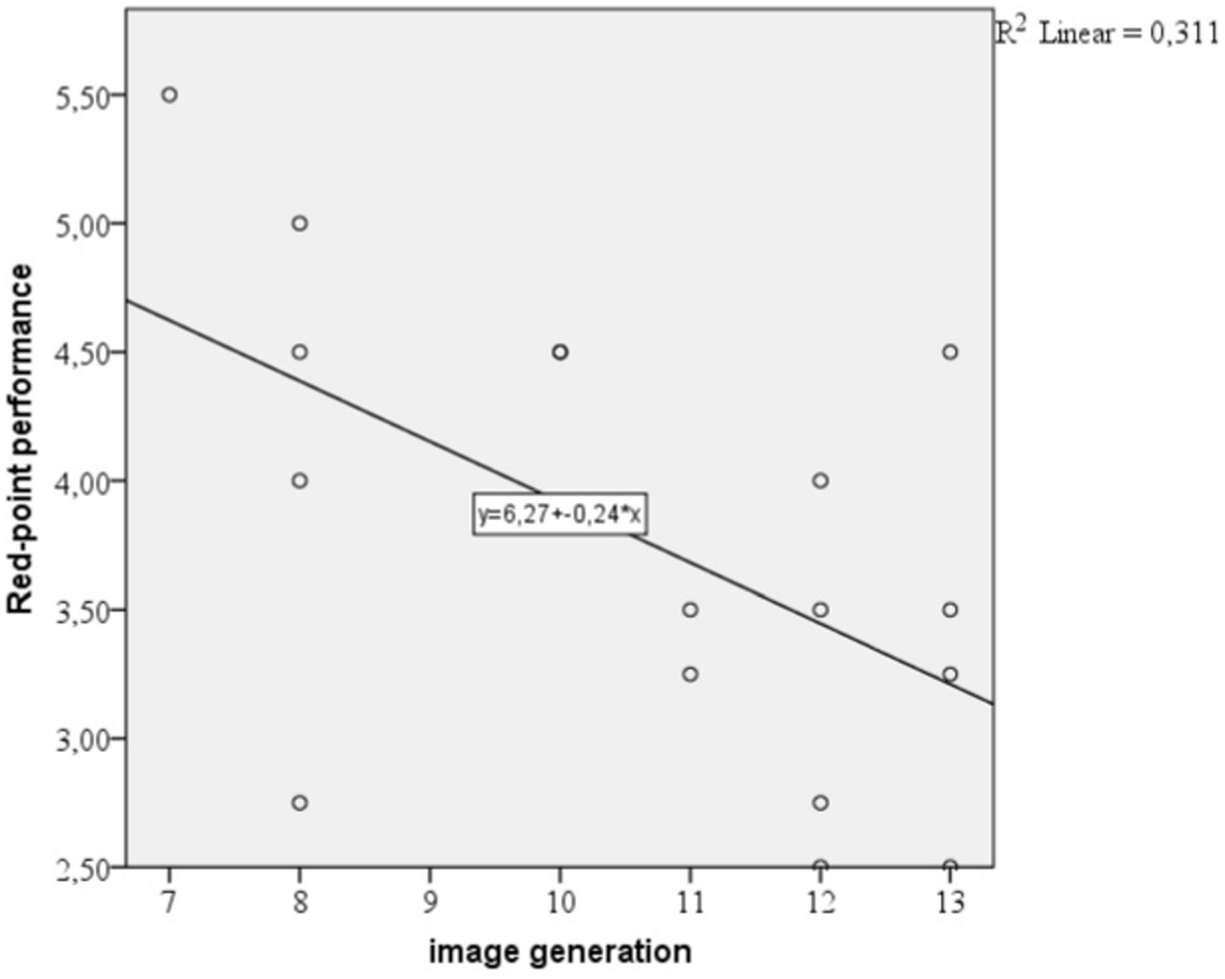
Scatterplot explaining simple linear regression between image generation and red point performance.

We can see the same result on the scatterplot showed in Figure 5, that is a simple regression model where the independent variable is image generation and the dependent variable is red-point performance.

## Discussion

The first conclusion of our study is that experience positively corrrelates with performance in youth climbing. Additionally, climbing performance can be predicted by experience. Climbing experience explained 42.7% of the variance of on-sight performance, and 49.5% of the variance of red-point performance. These results prove the same point as other previous studies such the one conducted by Birkett (1988) who explained that climbing skills are dependent on experience and technique. Other studies explained that the more experienced a climber is, the smaller force they apply on a hold, the shorter the contact time is and the larger the coefficient of friction is (Saul et al., 2019). Also, elite climbers seem to be more focused than an inexperienced climber demonstrated by fewer errors in complex reaction time test (Magiera et al., 2013). Saul et al. (2019) concluded that, similar to various different sports, experience is a key factor of success in climbing. Additionally, our study explained that experience predicts better red-point performance than on-sight performance with almost 7%. This result can be explained by the fact that red-point climbing is less demanding than on-sight from physiological and psychologycal points of view (Limonta et al., 2020). On-sight climbing requires greater levels of cognitive skills, such as route intepretation strategies, spatial orientation, motric memory, problem-solving skills, but also greater levels of psychological skills such as stress management, risk management, coping anxiety (Byrne and Mueller, 2014). Limonta et al. (2020) demonstrated that for a red-point climb, the ascent is faster, smoother and less demanding physiologically (with lower levels of lactate) and psychologically (lower cognitive anxiety, lower somatic anxiety, higher self-confidence).

From the multiple regression analyses, our study explained the following: for on-sight performance, the independent variables (climbing experience and image generation) predict 42.4% of the variance of dependent variable. So there is a difference of 0.5% which is due to image generation variable. From the regression equation image generation variable has a negative coefficient which explains that when image generation is higher, the on-sight performance is lower. For red point performance, the independent variables (climbing experience and image generation) predict 48% of the variance of dependent variable. So there is a difference of 1.5% which is due to image generation variable. From the regression equation image generation variable has a negative coefficient which explains that when image generation is higher, the red-point performance is lower.

Our study highlights that the cognitive ability measured with the image generation test has a negative effect on climbing performance in youth climbers, with a higher influence on red-point performance than on on-sight performance. Whilst the influence is negative, comparing to other cognitive skills that we measured, we can conclude that image generation influences performance. The image generation is part of the spatial orientation skills and measured the athlete’s ability to generate new mental images by combining other previously memorized. Our result that image generation negatively influences performance in climbing can be explained by the fact that when a climber has to mentally simulate the plan for ascending the route they have to find the optimal solution for passing the crux. When finding solutions, they search in their mind for multiple ways for passing the crux and have to choose only one that will lead them to success. Finding a solution means searching in their mind previous cruxes from previous climbed routes similar to the one they have and use their motric memory to successfully choose the final approach. This is the reason why climbing skills are in relation with technique (Birkett, 1988), because with greater technique comes more climbed routes and more muscle memory formed on previous climbs. When a climber has a high image generation, they have a higher ability to generate new mental images which can lead to viewing more approaches for passing a crux. Initially, this can be seen as a good skill, but when climbing already means multiple permutations of movements in athlete’s mind over on an exhaustation physical and psychical state, can lead to failure. In conclusion, a high level of image generation ability can lead to viewing more approaches for passing the crux, but in a moment of physical and mental breakdown, can lead to failure. Previous studies (Harm, 2019) explained that, when generating images, the decoder needs to determine what kind of image to generate before starting generation-exactly like in route previewing. The authors (Harm, 2019) explained that in climbing the route is analyzed in items such as”number of holds,”” distance between holds,”“holds’ placement on the board” and the climber has to train his mental ability to memorize these items and map the holds in their mind in order to choose the right approach for climbing which will lead them to succes. The ability of choosing the right approach for passing a crux in a route from multiple possible solutions is believed to be a part of the tactical preparation (Trifu et al., 2021).

In on-sight performance the orientation of the athlete in relation to the route, the wall, the rock, the characteristics of the route and the positioning of the holds is much more important. In on-sight performance, the athlete sees a route for the first time, without any previous practice or knowledge about the holds, the wall or the plan for ascending. The climber can not obtain additional information about the grips, the movements or the approach plan (Schweizer and Furrer, 2007). The climber must calculate the distances between the holds and orient themselves to find the resting places, the places where to secure the carabiniers, where the crux is on the route and how to position themselves to solve it. At the very first attempt, the athlete must orient as best as possible on a route seen for the first time, which requires skills related to kinesthetic orientation, proprioception, body awareness and orientation towards the environment. On-sight climbing is usually used in competitions, so elite climbers have to increase the importance of cognitive training, with emphasis on spatial orientation and reactivity.

In red-point climbing, the athlete must try several plans for ascending the route and use motor memory to decide upon the successful approach in the past. When climbing red-point, the athlete practices multiple times the route. Practice allows climbers to rehearse movement sequences, evaluate fall risks and likelihood of success or failure (Harm, 2019). Physical pre-practice is not permitted in competition, where the performance is labeled as on-sight. For red-point performance, in order to achieve the top after several attempts, the athlete tries to find the optimal way for solving the crux and then top the route. There are climbers that try routes for years until they manage to succed.

The importance of cognitive skills in climbing is supported by several studies (Limonta et al., 2020) according to which route intepretation and movement sequence recall are two essential cognitive skills for performance optimization. The same idea is accepted by Sanchez et al. (2012), who explain that the ability to memorize (information acquired during previous attempts) is crucial in red-point climbing. The importance of spatial orientation is also noted by previous studies (Seifert et al., 2017), especially in route previewing. The output climbing performance is also influenced by the type of previewing: normal previewing and video-model previewing seem to increase the opportunities to successfully climb a bouldering route (Morenas et al., 2021). Climbers do not focus on the same aspects of the route and have different interpretative strategies. Some climbers mentally map the sequencing of the holds and determine the spatial objectives useful for climbing, while others focus on the body weight redistribution and upper and lower limb coordination (Limonta et al., 2020). Expert climbers are more focused on the functional aspects of the ascent (how the holds are connected and how to move with the entire body between them), while beginners focus on the structural characteristics of the holds (their shape, size, orientation) (Boschker et al., 2002). Limonta et al. (2020) explain that differences in climbing skill levels correspond to differences in visual perception and memory. These gnostic processes are trainable skills.

Our study sought to identify the cognitive variables predicting on-sight and red-point performance. We started from the idea of others authors (Limonta et al., 2020) claiming that the way a climber thinks before a climb differs between an on-sight ascent and a red-point ascent. When climbers make an on-sight attempt, they push their limits beyond their current ability levels and are unlikely to succeed without falling (Aras and Akalan, 2014). When they pass over the on-sight attempt, they begin to analyze the moves and the sequences of moves, focusing on the crux or the personal harder part of the route. After a better analysis of the route, they will attempt a red-point ascent, making use of the information and physical experience acquired during previous attempts. The ability of recalling the moves and the correct sequence to pass a crux but also incorporating motor learning skills into the ascent is essential to reduce the number of attempts to reach the top (Sanchez et al., 2019). When an on-sight climb is unsuccessful, the climber uses the route preview for the next attempts by mentally rehearsing the move sequences and reprogramming the distribution of effort without physical and mental stress. After the failure of an on-sight climb, the athlete reduces the number of attempts for red-pointing by optimizing the route preview. This is a skill important in lead and bouldering, in both training and competition, because athletes need to preserve their energy by minimizing the number of attempts (White and Olsen, 2010). Our results also have implications for the need for cognitive training in sport climbing, especially in competitive climbing. The effects of cognitive training in the sport field are often discussed in sport psychology (Veraksa et al., 2012). There is no doubt that sports performance is linked to cognitive and perceptual skills but also motor and physical skills (Grusko and Leonov, 2014).

The study has some limitations. Cross-sectional design cannot extrapolate about the causal relationship between the variables. The number of participants was relatively small due to inclusion criteria, but we limited to analyzing the Romanian elite youth climbers. On the other hand, the instruments that we used are not measuring specific cognitive skills from climbing; several studies should focus on developing a questionnaire that measures the exact cognitive skills that a climber needs in a climbing format setting. Another limitation is the variability of the group ages: we analyzed climbers aged between 14 and 19 years and at these ages there are major cognitive leaps from year to year. The cognitive development of a 14-year-old athlete may be slightly different from that of a 19-year-old athlete, Fischer (2008) explaining that they transition from abstract mappings to abstract systems. Also, the fact that we analyzed both male and female climbers might have influenced both the cognitive measurement and climbing performance. Rubia et al. (2013) explained that females from their study group were more efficient than males in tasks that required selective attention and conductive reasoning, while males were more efficient in tasks based on visual–spatial processing. Brain growth in women starts and ends earlier than in men, peaking at 10.5 years old, while in men the peak is around 14.5 years old (Lenroot et al., 2007). Lastly, another limitation is related to the athletes’ school background, each of them being from a different school and each school being at a different level of educating their students: some climbers are students in a sports high school (3), some of them are students in normal schools from their cities (3), others (7) are in elite high schools from their cities and others (4) are freshmen in universities. The school background variable may have influenced the athletes’ cognitive results.

## Data availability statement

The raw data supporting the conclusions of this article will be made available by the authors, without undue reservation.

## Ethics statement

The studies involving human participants were reviewed and approved by the Ethical Committee of the National University of Sport and Physical Education, Bucharest, Romania. Written informed consent to participate in this study was provided by the participants’ legal guardian/next of kin.

## Author contributions

All authors listed have made a substantial, direct, and intellectual contribution to the work and approved it for publication.

## Conflict of interest

The authors declare that the research was conducted in the absence of any commercial or financial relationships that could be construed as a potential conflict of interest.

## Publisher’s note

All claims expressed in this article are solely those of the authors and do not necessarily represent those of their affiliated organizations, or those of the publisher, the editors and the reviewers. Any product that may be evaluated in this article, or claim that may be made by its manufacturer, is not guaranteed or endorsed by the publisher.

## Footnotes

^1^ https://frae.ro/despre/membri/cluburi-afiliate

^2^ https://www.frae.ro/sporturi/statistica-frae

## References

[ref1] Aras D. Akalan C. (2014). The effect of anxiety about falling on selected physiological parameters with different rope protocols in sport rock climbing. J. Sports Med. Phys. Fit. 54, 1–8. 24445539

[ref2] Ascii F. H. Demirhan G. Dinc¸ S. C. (2007). Psychological profile of Turkish rock climbers: an examination of climbing experience and route difficulty. Percep. Motor Skills 104, 892–900. doi: 10.2466/pms.104.3.892-900, PMID: 17688145

[ref3] Attali M. Saint-Martin J. (2016). Outdoor physical education in French schools during the twentieth century. J. Adv. Educ. Outdoor Learn. 17, 148–160. doi: 10.1080/14729679.2016.1242082

[ref4] Birkett B. (1988). Techniques in Modern Rock and Ice Climbing. London: A.N.C. Black

[ref5] Booth J. Marino F. Hill C. Gwinn T. (1999). Energy costs of sport rock climbing in elite performers. Br. J. Sports Med. 33, 14–18. doi: 10.1136/bjsm.33.1.14, PMID: 10027051 PMC1756138

[ref6] Boschker M. S. Bakker F. C. Michaels C. F. (2002). Memory for the functional characteristics of climbing walls: perceiving affordances. J. Mot. Behav. 34, 25–36. doi: 10.1080/00222890209601928, PMID: 11880247

[ref7] Byrne R. Mueller F. (2014). Designing digital climbing experiences through understanding rock climbing motivation. eds. Pisan Y.. ICEC 2014. LNCS 8770, 92–99. doi: 10.1007/978-3-662-45212-7_12

[ref8] Draper N. Dickson T. Blackwell G. Fryer S. Priestley S. Winter D.. (2011a). Self-reported ability assessment in rock climbing. J. Sports Sci. 29, 851–858. doi: 10.1080/02640414.2011.565362, PMID: 21491325

[ref9] Draper N. Dickson T. Fryer S. Blackwell G. (2011b). Performance differences for intermediate rock climbers who successfully and unsuccessfully attempted an indoor sport climbing route. Int. J. Perform. Anal. Sport 11, 450–463. doi: 10.1080/24748668.2011.11868564

[ref10] Draper N. Giles D. Schöffl V. Konstantin Fuss F. Watts P. Wolf P.. (2015). Comparative grading scales, statistical analyses, climber descriptors and ability grouping: international rock climbing research association position statement. Sports Technol. 8, 88–94. doi: 10.1080/19346182.2015.110708

[ref11] Draper N. Jones G. A. Fryer S. Hodgson C. Blackwell G. (2008). Effect of an on-sight lead on the physiological and psychological responses to rock climbing. J. Sports Sci. Med. 1, 492–498. PMC3761930 24149956

[ref12] Fischer K. W. (2008). “Dynamic cycles of cognitive and brain development: measuring growth in mind, brain, and education,” in The Educated Brain. eds. Battro A. M. Fischer K. W. Léna P. (Cambridge: Cambridge University Press)

[ref13] Grusko A. I. Leonov S. V. (2014). The usage of eye-tracking technologies in rock-climbing. Procedia Soc. Behav. Sci. 146, 169–174. doi: 10.1016/j.sbspro.2014.08.075

[ref14] Harm V D. (2019). Climbing creativity: teaching a neural network to create routes for the Moonboard training board. Bachelor’s Thesis, Available at: https://theses.ubn.ru.nl/handle/123456789/10867 (Accessed June 10, 2022).

[ref15] Horst EJ. (2003). Training for Climbing. Guilford, CT: Globe Pequot Press.

[ref17] Kascenska J. DeWitt J. Roberts T. (1992). Fitness guidelines for rock climbing students. JOPERD 63, 73–79. doi: 10.1080/07303084.1992.10604140

[ref19] Kline R. B. (2011). Principles and Practice of Structural equation Modeling (5th ed). New York: The Guilford Press.

[ref20] Lenroot R. K. Gogtay N. Greenstein D. K. Wells E. M. Wallace G. L. Clasen L. S.. (2007). Sexual dimorphism of brain developmental trajectories during childhood and adolescence. NeuroImage 36, 1065–1073. doi: 10.1016/j.neuroimage.2007.03.053, PMID: 17513132 PMC2040300

[ref21] Limonta E. Fanchini M. Rampichini S. Cé E. Longo S. Coratella G.. (2020). On-sight and red-point climbing: changes in performance and route-finding ability in male advanced climbers. Front. Psychol. 11:902. doi: 10.3389/fpsyg.2020.00902., PMID: 32547440 PMC7271724

[ref23] Lutter C. Tischer T. Schöffl V. R. (2021). Olympic competition climbing: the beginning of a new era—a narrative review. Br. J. Sports Med. 55, 857–864. doi: 10.1136/bjsports-2020-102035, PMID: 33036996

[ref24] Magiera A. Roczniok R. Maszczyk A. Czuba M. Kantyka J. Kurek P. (2013). The structure of performance of a sport rock climber. J. Hum. Kinet. 36, 107–117. doi: 10.2478/hukin-2013-0011, PMID: 23717360 PMC3661882

[ref25] Mermier C. Robergs R. McMinn S. Heyward V. (1997). Energy expenditure and physiological responses during indoor rock climbing. Br. J. Sports Med. 31, 224–228. doi: 10.1136/bjsm.31.3.224, PMID: 9298558 PMC1332525

[ref26] Morenas J. Luis del Campo V. López-García S. Flores L. (2021). Influence of on-sight and flash climbing styles on advanced climbers’ route completion for bouldering. Int. J. Environ. Res. Public Health 18:12594. doi: 10.3390/ijerph182312594, PMID: 34886320 PMC8657215

[ref27] Morrison A. B. Schöffl V. R. (2007). Review of the physiological responses to rock climbing in young climbers. Br. J. Sports Med. 41, 852–861. doi: 10.1136/bjsm.2007.034827, PMID: 18037632 PMC2658987

[ref28] Naderi K. Takatalo J. Lipsanen J. Hamalainen P. (2018). “Computer-aided imagery in sport and exercise: a case study of indoor wall climbing”. in *Graphics Interface Conference*.

[ref29] Rhea M. R. Hunter R. L. Hunter T. D. (2006). Competition modeling of American football: observational data and implications for high school, collegiate, and professional player conditioning. J. Strength Cond. Res. 20, 58–61. doi: 10.1519/00124278-200602000-00010, PMID: 16503692

[ref31] Rubia K. Lim L. Ecker C. Halari R. Giampietro V. Simmons A.. (2013). Effects of age and gender on neural networks of motor response inhibition: from adolescence to mid-adulthood. NeuroImage 83, 690–703. doi: 10.1016/j.neuroimage.2013.06, PMID: 23845427

[ref16] Sanchez X. Jones G. (2016). “Psychological processes in the sport of climbing,” in The science of climbing and mountaineering. Routledge Research in Sport and Exercise Science. eds. Ludovic S. Peter W. Andreas S. (Abingdon, UK: Routledge), 244–256.

[ref32] Sanchez X. Lambert P. Jones G. Llewellyn D. J. (2012). Efficacy of pre-ascent climbing route visual inspection in indoor sport climbing. Scand. J. Med. Sci. Sports 22, 67–72. doi: 10.1111/j.1600-0838.2010.01151.x, PMID: 20561271

[ref33] Sanchez X. Torregrossa M. Woodman T. Jones G. Llewellyn D. J. (2019). Identification of parameters that predict sport climbing performance. Front. Psychol. 10:1294. doi: 10.3389/fpsyg.2019.01294, PMID: 31214092 PMC6554989

[ref35] Saul D. Steinmetz G. Lehmann W. Schilling A. F. (2019). Determinants for success in climbing: a systematic review. J. Exerc. Sci. Fit. 17, 91–100. doi: 10.1016/j.jesf.2019.04.002, PMID: 31193395 PMC6527913

[ref36] Schweizer A. Furrer M. (2007). Correlation of forearm strength and sport climbing performance. Isokinet. Exerc. Sci. 15, 211–216. doi: 10.3233/IES-2007-0275

[ref37] Seifert L. Cordier R. Orth D. Courtine Y. Croft J. L. (2017). Role of route previewing strategies on climbing fluency and exploratory movements. PLoS One 12:e0176306. doi: 10.1371/journal.pone.0176306, PMID: 28441425 PMC5404847

[ref39] Trifu A. Stanescu M. Pelin F. (2021). Features of tactical and psychological training models in sports climbing at youth level. J. Educ. Sci. Psychol. 11, 153–162. doi: 10.51865/JESP.2021.1.14

[ref40] Veraksa A. N. Gorovaya A. E. Leonov S. V. Pashenko A. K. Fedorov V. V. (2012). The possibility of using sign and symbolic tools in the development of motor skills by beginning soccer players. Psychol. Russia 5, 473–497. doi: 10.11621/pir.2012.0030

[ref42] Watts P. B. (2004). Physiology of difficult rock climbing. Eur. J. App. Phys. 91, 361–372. doi: 10.1007/s00421-003-1036-7, PMID: 14985990

[ref43] Watts P. B. Martin D. T. Durtschi S. (1993). Anthropometric profiles of elite male and female competitive sport rock climbers. J. Sports Sci. 11, 113–117. doi: 10.1080/02640419308729974, PMID: 8497013

[ref44] White D. J. Olsen P. D. (2010). A time motion analysis of bouldering style competitive rock climbing. J. Strength Cond. Res. 24, 1356–1360. doi: 10.1519/JSC.0b013e3181cf75bd, PMID: 20386481

[ref45] Williams E. Taggert P. Carruthers M. (1978). Rock climbing: observations on heart rate and plasma catecholamines and the influence of oxprenolol. Br. J. Sports Med. 12, 125–128. doi: 10.1136/bjsm.12.3.125, PMID: 719320 PMC1859664

[ref46] Yogev-Seligmann G. Rotem-Galili Y. Mirelman A. Dickstein R. Giladi N. Hausdorff J. M. (2010). How does explicit prioritization alter walking during dual-task performance? Effects of age and sex on gait speed and variability. Phys. Ther. 90, 177–186. doi: 10.2522/ptj.20090043, PMID: 20023000 PMC2816029

